# Cell-type specific light-mediated transcript regulation in the multicellular alga *Volvox carteri*

**DOI:** 10.1186/1471-2164-15-764

**Published:** 2014-09-06

**Authors:** Arash Kianianmomeni

**Affiliations:** Department of Cellular and Developmental Biology of Plants, University of Bielefeld, Universitätsstr. 25, D-33615 Bielefeld, Germany

**Keywords:** Cell types, Light quality, Gene expression, Photoreceptors, Green algae

## Abstract

**Background:**

The multicellular green alga *Volvox carteri* makes use of none less than 13 photoreceptors, which are mostly expressed in a cell-type specific manner. This gives reason to believe that trasncriptome pattern of each cell type could change differentially in response to environmental light. Here, the cell-type specific changes of various transcripts from different pathways in response to blue, red and far-red light were analyzed.

**Results:**

In response to different light qualities, distinct changes in transcript accumulation of genes encoding proteins involved in chlorophyll and carotenoid biosynthesis, light-harvesting complexes, circadian clock and cell cycle control were observed. Namely, blue light tends to be effective to accumulate transcripts in the somatic cells; while red light leads to accumulate transcripts predominantly in the reproductive cells. Blue light also induced marked accumulation of two components of circadian rhythms only in the somatic cells, indicating that these clock-relevant components are affected by blue light in a cell-type specific manner. Further, we show that photosynthetic associated genes are regulated distinctly among cell types by different light qualities.

**Conclusion:**

Our results suggest that *Volvox* uses different sophisticated cell-type specific light signaling pathways to modulate expression of genes involved in various cellular and metabolic pathways including circadian rhythms and photosynthesis in response to environmental light.

**Electronic supplementary material:**

The online version of this article (doi:10.1186/1471-2164-15-764) contains supplementary material, which is available to authorized users.

## Background

The photosynthetic organisms such as free swimming microalgae use light signals to modulate a wide variety of physiological and cellular responses including sexual life cycle, circadian clock, cellular differentiation, cell cycle, nitrogen and lipid metabolism [1–4]. A sophisticated light-sensing system including various photoreceptors has been developed during evolution to monitor changes in the ambient light environment (quality, quantity, direction and duration) towards adjust growth and development (reviewed in [5–7]). Perception of light by -some of- photoreceptors activates signal transduction cascades which lead to regulate gene expression patterns during development or in response to different light signals [8, 9]. These light-induced signal transduction pathways orchestrate the expression of downstream genes responsible for various physiological processes including circadian clock, chlorophyll and carotenoid biosynthesis [10, 11].

**Figure 1:**
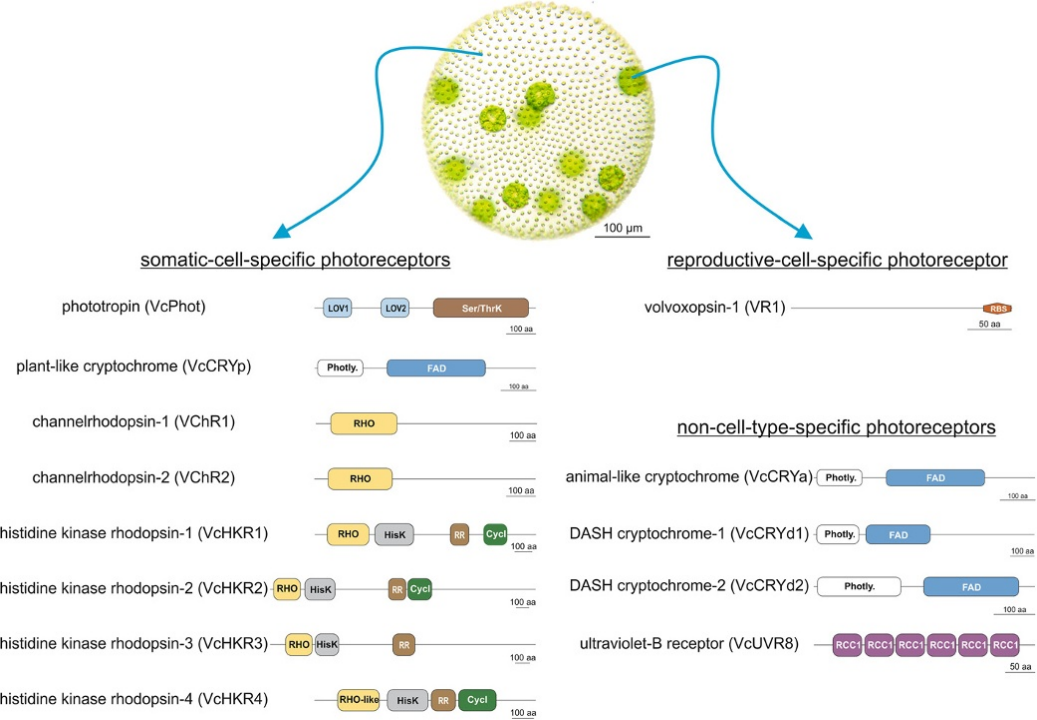
***Volvox*** **photoreceptors.** Photograph of multicellualr *Volvox* and domain composition of its photoreceptors according to the Pfam database. Two different cell types, i.e., large dark green reproductive cells and small pale biflagellate somatic cells are located below and at the surface, respectively. Photoreceptors are categorized in three groups, i.e., somatic-, reproductive- and non-cell-specific photoreceptors, on the basis of their cell-type specific transcript levels (Additional file 2: Figure S2 and Additional file 4: Figure S3). Proteins are drawn approximately to scale. Domain abbreviations are photly. (photolyase domain), FAD (flavin adenine dinucleotide binding domain), LOV (Light-oxygen-voltage), Ser/ThrK (serine/threonine kinase), RBS (retinal binding site), RHO (rhodopsin), HisK (histidine kinase), RR (response regulator), Cycl (adenylate/guanylate cyclase domain), RCC1 (regulator of chromosome condensation).

Four major types of photoreceptors, i.e., phototropins, cryptochromes, rhodopsisns and UV-B photoreceptors, have been identified so far in the genome of volvocine algae, a group of chlorophytes including unicellular *Chlamydomonas reinhardtii* (hereafter *Chlamydomonas*) and multicellular *Volvox carteri* (hereafter *Volvox*) (reviewed in [6]). Although *in vivo* functions of some of these photoreceptors have been investigated in some detail in the unicellular *Chlamydomonas* [3, 10, 12–14], little is known about the molecular background of light reception mechanisms in the multicellular *Volvox*. However, the confusing maze behind individual activities of photoreceptors in the multicellular *Volvox* could contribute to understand the link between light and complex light-affected developmental processes such as cellular differentiation [2], which have been required for the evolutionary transition from unicellular organisms into a multicellular one [15, 16]. *Volvox* is one of the simplest multicellular organisms composed of only two cell types, 2000–4000 biflagellate motile, terminally differentiated somatic cells, which build a monolayer at the surface of a spheroid, and around 16 much larger immotile reproductive cells (so-called gonidia), which lie just below the somatic cell sheet; the cells are embedded in a transparent sphere of glycoprotein-rich, extracellular matrix (ECM) (Figure 1) [17, 18].

We recently have shown that *Volvox* photoreceptors are expressed in a cell-type-specific manner. Eight photoreceptors, i.e., a phototropin, a plant-like cryptochrome, channelrhodopsin-1 and -2 and four histidine kinase rhodopsins, highly express in the somatic cells [6, 16, 19], while only one photoreceptor, i.e. volvoxopsin-1, has been found to predominantly express in the reproductive cells [16, 20] (Figure 1). These evidences strongly suggest that distinct cell type-specific light signaling pathways orchestrate gene expression in each cell type. Here, we investigated the cell type-specific changes in transcript accumulation of genes involved in downstream light signaling pathways. First, a diversified set of genes encoding components of light-harvesting system, carotenoid biosynthesis, tetrapyrrole biosynthesis, nitrogen metabolism, circadian clock and cell cycle were selected (Table 1). Further, we examined the light-responsive expression of these genes in each cell types after exposure to blue, red and far-red lights. The results showed that the changes in transcript level underlined distinct light signaling pathways in each cell type. Moreover, we found that some of the selected genes become rapidly upregulated after the somatic cells were exposed to blue light, whereas the same genes were induced by red light in the reproductive cells. Our results show that different light qualities lead to cell-type specific expression or repression of genes, indicating the existence of different cell type specific light signaling pathways required for physiological and developmental adaptation to environmental light.

**Table 1:** **Candidate genes chosen for analysis of cell-type specific change of transcript accumulation under blue, red and far-red light in** ***Volvox***

Gene	Description/Function	Accession Number/Reference	Percentage of sequence identity to the homologous protein from the closely related alga ***Chlamydomonas***	Accession number of used homolog for sequence comparison/Reference	Changes in transcript in response to light/References
*LHCBM6*	Chloropyll a-b binding protein of LHCII type I/a major LHCII polypeptide present in the trimeric antenna complexes of PS II	XM_002959515/[21]	93.3% identity in 253 aa overlap	EDP01611/[22]	Blue, red and far-red light/[10, 11, 23]
*LHL4*	Lhc-like protein Lhl4/distant relative of light-harvesting Chla/b protein	XP_002956040/[21]	64.1% identity in 284 aa overlap	BAD67138/[24]	Blue, green and red light/[25]
*CHLD*	Magnesium chelatase subunit D, chloroplast precursor/involved in chlorophyll biosynthesis	XP_002956151/[21]	93.3% identity in 704 aa overlap	EDP07156/[22]	White, blue and red light/[10, 26]
*GSA*	Glutamate-1-semialdehyde 2,1-aminomutase/key enzyme for chlorophyll synthesis	XP_002950034/[21]	92.2% identity in 464 aa overlap	Q39566/[27]	Blue, red and far-red light/[10, 11, 28]
*POR*	Protochlorophyllide reductase/involved in protochlorophyllide and chlorophyll biosynthesis	XP_002950278/[21]	81.1% identity in 392 aa overlap	Q39617/[29]	Blue and red light/[10]
*ALAD*	Delta-aminolevulinic acid dehydratase/key enzyme required for early steps in chlorophyll biosynthesis	XP_002946379/[21]	91% identity in 388 aa overlap	Q42682/[30]	White, blue and green light/[11, 28]
*OEE*	Oxygen evolving enhancer protein 1/part of the oxygen evolving complex of photosystem II	XP_002954867/[21]	87% identity in 291 aa overlap	P12853/[31]	White light/[11]
*RB60*	Disulfide isomerase RB60/part of a complex that regulates the translation of the chloroplast-encoded *psbA*	EFJ41881/[21]	71% identity in 532 aa overlap	AAC49896/[32]	White and red light/[33]
*RB38*	RNA-binding protein RB38/part of a complex that regulates the translation of the chloroplast-encoded *psbA*	XP_002953456/[21]	53.4% identity in 371 aa overlap	AAM76787/[34]	White, blue and red/[33]
*PDS*	Phytoene desaturase/key enzyme for carotenoids synthesis	XP_002948155/[21]	85.3% identity in 572 aa overlap	EDP05305/[22]	Blue and red light/[10, 11, 35]
*PSY*	Phytoene synthase/key enzyme involved in the first step of the carotenoids biosynthetic pathway	XP_002956783/[21]	86.3% identity in 379 aa overlap	EDO97702/[22]	White and blue light/[11, 35, 36]
*CDKB1*	Plant specific cyclin dependent kinase/involved in the regulation of the cell cycle	XP_002947156/[21]	95.7% identity in 322 aa overlap	EDO97594/[22]	Blue and red light/[10]
*CRB1*	C1 subunit of the circadian RNA-binding protein CHLAMY1/associated with the circadian clock	XP_002957962/[21]	57.9% identity in 482 aa overlap	EDP08399/[22]	Blue and red light/[10]
*CRB3*	C3 subunit of the circadian RNA-binding protein CHLAMY1/associated with the circadian clock	XP_002946862/[21]	85.2% identity in 392 aa overlap	EDP06114/[22]	Blue and red light/[10]
*ACDA*	Guanylyl and adenylate cyclase family member/cAMP or cGMP production	ABM47321/[37]	63.6% identity in 154 aa overlap	XP_001702503/[22]	---------------
*CA*	Carbonic anhydrase/key enzyme involves in involved in carbon metabolism	XP_002951242/[21]	50.8% identity in 374 aa overlap	BAA14232/[38]	White, blue and red light/[36, 39]
*GLN1*	Glutamine synthetase/key enzyme in nitrogen metabolism	XP_002956198/[21]	92.1% identity in 382 aa overlap	U46207/[40]	White, blue and red light/[10, 40]
*FBP*	Fructose-1,6-bisphosphatase/key enzymes involved in gluconeogenesis and the Calvin cycle	XP_002948170 [21]	88% identity in 415 aa overlap	XP_001690872/[22]	White light/[36]
*RPE*	Ribulose phosphate-3-epimerase/key enzymes involved in the Calvin cycle	Photozyme ID: Vocar20000806m.g/[21]	90.2% identity in 264 aa overlap	XP_001691071/[22]	White light/[36]

## Methods

### Strain and culture conditions

Cultures of *Volvox carteri* f. *nagariensis* female strain Eve10 [41] were maintained in standard *Volvox* medium [42] at 28°C in a cycle of 8 h dark/16 h cool fluorescent white light at an average of ~100 μmol photons m^-2^ s^-1^ photosynthetically-active radiation (PAR). The reproductive and somatic cells were separated three hours before initiation of cleavage division (Additional file 1: Figure S1A) using Dounce homogenizer as described previously [37].

### Light treatments

To analyze cell-type specific light-mediated gene expression, the isolated reproductive and somatic cells were incubated for 26 hours in the dark before exposure to the test light of specific wavelengths for 1 hour (Additional file 1: Figure S1B). Light treatments were performed using LEDs of specific wavelengths with following equipment and settings: for blue light, Luxeon Rebel High Power Blue LED (Philips Lumileds), peak at 470 nm (with a typical spectral half-width of 20 nm) and a photon fluence rate of 15 μmol photons m^-2^ s^-1^; for red light, Luxeon Rebel High Power Deep Red LED (Philips Lumileds), peak at 655 nm (with a typical spectral half-width of 20 nm) and a photon fluence rate of 15 μmol photons m^-2^ s^-1^; for far-red light, High Efficacy Far Red LED (LED Engin), peak at 735 nm (with a typical spectral half-width of 25 nm) and a photon fluence rate of 15 μmol photons m^-2^ s^-1^ (Additional file 2: Figure S2). The light fluence rates were measured using a LI-250A light meter (Li-COR) and a red light meter (Model 9.6 Visible Red Light Meter, Solartech inc.).

### RNA isolation

Total RNA was extracted from all samples using TRI Reagent (Sigma-Aldrich, St. Louis, MO) as described previously [19]. The extracted RNA was dissolved in RNase-free water and stored at -70°C. The integrity and size distribution of total RNA was checked by denaturing agarose-formaldehyde gel electrophoresis. The quantity and quality of extracted RNA were determined spectrophotometrically using an Ultrospec 2100 proUV/Visible Spectrophotometer (GE Healthcare) at 260 and 280 nm.

### Quantitative one-step real-time RT-PCR and data analysis

Primers were designed to amplify cDNA fragments with 109 to 144 bp in length (Additional file 3: Table S1). 1 μg total RNA from each cell type (dark-adapted and after light treatments) was treated with 5 units DNaseI (Promega, Madison, WI) in DNase-I buffer (20 mM Tris, pH 8.4, 2 mM MgCl_2_, 50 mM KCl) in a total volume of 10 μl at 37°C for 10 min to remove contaminating DNA within the RNA preparation. The reaction was stopped by the addition of 1 μl 25 mM EDTA and incubation at 65°C for 10 min. The RNA concentration was measured before and after DNAse-I treatment to ensure that the same RNA amount from different cell types were used in all reactions. The real-time RT-PCR was performed on a CFX96TM real-time PCR detection system (Bio-Rad) using the SensiFAST SYBR One-Step Kit (Bioline). All reactions contained 300 ng DNase-I-treated template RNA in a total volume of 20 μl. The reactions were incubated for 30 min at 50°C for cDNA synthesis followed by 2 min incubation at 95°C for initial Tag polymerase activation. The reactions were then subjected to 40 cycles of amplification, which consisted of a denaturing step at 95°C for 5 s, an annealing step at 57°C for 10 s and an extension step at 72°C for 5 s. All real-time RT-PCR experiments were carried out in triplicate from two different biological samples together with controls lacking RT or template to detect potential DNA contaminations. The relative expression level has been calculated using the 2^-∆∆Ct^ as described previously [37, 43]. *RACK1*, which has previously been used for normalization of light-dependent gene expression in the closely related algae *Chlamydomonas reinhardtii* [10, 11] and shows constitutive expression in both reproductive and somatic cells, was used for normalization of cell type specific light-mediated gene expression data.

## Results

### Reproductive and somatic cells accumulate different photoreceptors

The study of *Volvox* photoreceptors was almost always accompanied by questions regarding their cell-type specific functions. This multicellular green alga makes use of no less than 13 photoreceptors, i.e., seven rhodopsin-like photoreceptors (*VR1*, *VChR1*, *VChR2*, *VcHKR1*, *VcHKR2*, *VcHKR3* and *VcHKR4*), one UV-B photoreceptor (*VcUVR8*), four crypochromes (*VcCRYa*, *VcCRYp*, *VcCRYd1* and *VcCRYd2*) and one phototropin (*VcPhot*) (Figure 1). In addition to the previous studies which showed that *VR1* and channelrhodopsins are highly expressed in the reproductive and somatic cells, respectively [19, 20], recent investigations revealed that most photoreceptors are expressed in a cell-type specific manner [6, 16] (Figure 1). These observations raise the question about the existence of different cell-type specific light signaling pathways in *Volvox*. To address this issue, we analyzed the cell-type specific change in the transcript accumulation of a set of genes which show light-induced changes in gene expression and are parts of downstream light signaling pathways [10, 11, 23, 25, 33, 35, 36, 39]. For this purpose, the reproductive and somatic cells were separated three hours before initiation of cleavage division, followed by 26 h of incubation in the dark before exposure to blue, red or far-red light (Additional file 1: Figure S1A-B). However, because the cell-type specific transcript level of photoreceptors depends on the developmental stages, we analyzed their transcript levels right after separation of cell types, i.e. three hours before initiation of cleavage division, and at the end of dark incubation step (Additional file 1: Figure S1A-B). The results were in accordance with the previously reported data [6, 16, 19, 20] and showed the same localization of cellular expression for *Volvox* photoreceptors (Additional file 4: Figure S3 and Additional file 5: Figure S4, Figure 1).

### *Volvox*genes encoding components of light-harvesting system, carotenoid and chlorophyll biosynthesis, circadian clock, cell cycle and diverse metabolic pathways

In order to examine whether distinct cell-type specific light signaling pathways are exist in *Volvox*, the impact of light quality on the expression of a variety of genes encoding components of light-harvesting system, carotenoid and chlorophyll biosynthesis, circadian clock, cell cycle and diverse metabolic pathways was investigated. Because of the absence of any report studying light-mediated gene expression in *Volvox*, candidate genes were selected based on available observations from other algal systems such as *Chlamydomonas* and *Phaeodactylum tricornutum* [10, 11, 26, 33, 35, 36]. The genes encoding components involved in light-harvesting system and chlorophyll biosynthesis include chloropyll a-b binding protein of LHCII type I (*LHCBM6*), a distant relative of light-harvesting chloropyll a-b protein (*LHL4*), magnesium chelatase subunit D (*CHLD*), glutamate-1-semialdehyde 2,1-aminomutase (*GSA*), protochlorophyllide reductase (*POR*), delta-aminolevulinic acid dehydratase (*ALAD*), oxygen evolving enhancer protein 1 (*OEE*), disulfide isomerase RB60 (*RB60*) and RNA-binding protein RB38 (*RB38*) (Table 1). Two genes encoding enzymes of carotenoid biosynthesis pathway, i.e., phytoene desaturase (*PDS*) and phytoene synthase (*PSY*), that have been shown to be controlled by light [10, 11, 35, 36] were also selected. Additional genes encoding clock and cell cycle relevant components including C1 and C3 subunits of the circadian RNA-binding protein CHLAMY1 (*CRB1* and *CRB3*) and a plant specific cyclin dependent kinase (*CDKB1*) were also analyzed (Table 1). Furthermore, genes encoding proteins involved in nitrogen metabolism (*GLN1*), carbon metabolisms (*CA* and *FBP*), pentose phosphate pathway (*PRE*) and a guanylyl and adenylate cyclase family member (Table 1) have been added to this study. None of these genes had been studied before in *Volvox* concerning blue, red and far-red light-dependent regulation.

### Cell-type specific changes in transcript accumulation of genes encoding photosynthetic related/associated components in response to blue and red light

**Figure 2:**
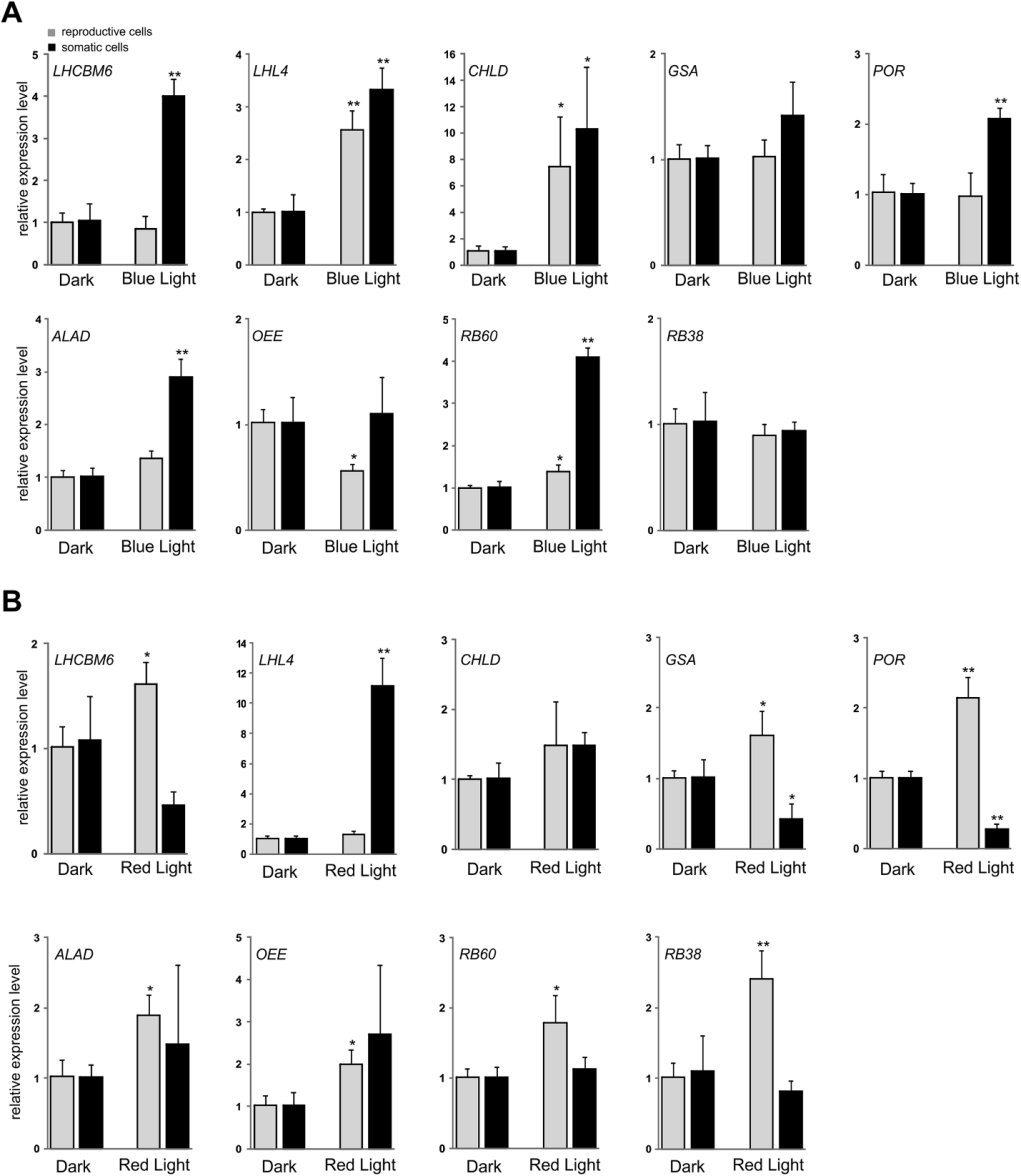
**Blue and red light-induced changes in transcript accumulation of genes encoding photosynthesis components.** The reproductive and somatic cells from vegetative algae were separated 3 h before initiation of cleavage divisions and incubated for 26 h in the dark before exposure to blue (**A**, 470 nm; 15 μmol photons m^-2^ s^-1^) and red (**B**, 655 nm; 15 μmol photons m^-2^ s^-1^) light for 1 hour (Additional file 1: Figure S1A-B). The transcript levels were calculated using the 2^-∆∆Ct^ method and *RACK1* as reference gene. Each experiment was performed in triplicate from at least two different biological samples. The data were averaged and statistically treated (t-test, *P < 0.05; **P < 0.01). Bars show means and standard deviations.

In plants including algae, red and blue light have been shown to regulate the expression of photosynthetic associated genes [25, 44, 45]. To examine the effect of blue and red light on cell-type specific transcript accumulation of genes encode components of photosynthetic apparatus and related regulatory elements in *Volvox*, the reproductive and somatic cells were exposed to the monochromic blue (470 nm) and red (655 nm) light of equal photon fluence rate of 15 photons m^-2^ s^-1^ for 1 h (Additional file 1: Figure S1). As shown in Figure 2, the transcript levels of *LHL4* and *CHLD* increased in response to blue light in both cell types (Figure 2A), while red light led to a marked increase in *LHL4* transcript (~11-fold) only in the somatic cells (Figure 2B). Following exposure of cell types to the blue light, the transcript of *LHCBM6* increased in the somatic cells, but remained unchanged in the reproductive cells (Figure 2A). In contrast, slightly elevated transcript level of *LHCBM6* was only observed in the reproductive cells in response to red light (Figure 2B). Furthermore, the level of *POR* transcript increased in response to the blue light in the somatic cells and, remarkably, decreased in those cells after exposure to the red light. However, only red light could elicit elevated expression of *POR* transcript in the reproductive cells (Figure 2A-B). Moreover, the transcript level of *OEE* increased in response to the red light in the reproductive cells, whereas blue light led to decrease its level in these cells. The detected changes in the transcript level of *OEE* in the somatic cells seem to be not statistically significant. The transcript of *RB60* increased in the somatic (remarkably) and reproductive (moderately) cells following blue light treatment, while red light led to elevated transcript level only in the reproductive cells (Figure 2A-B). These results show that blue and red light regulate the expression of genes encoding photosynthetic relevant components in both cell types in different ways, suggesting that cell-type specific light-induced signal transduction pathways underlying light-induced changes of photosynthetic related transcripts.

### Blue and red light-induced changes in transcript accumulation of genes encoding carotenoid biosynthesis, cell cycle and clock relevant components in the reproductive and somatic cells

**Figure 3:**
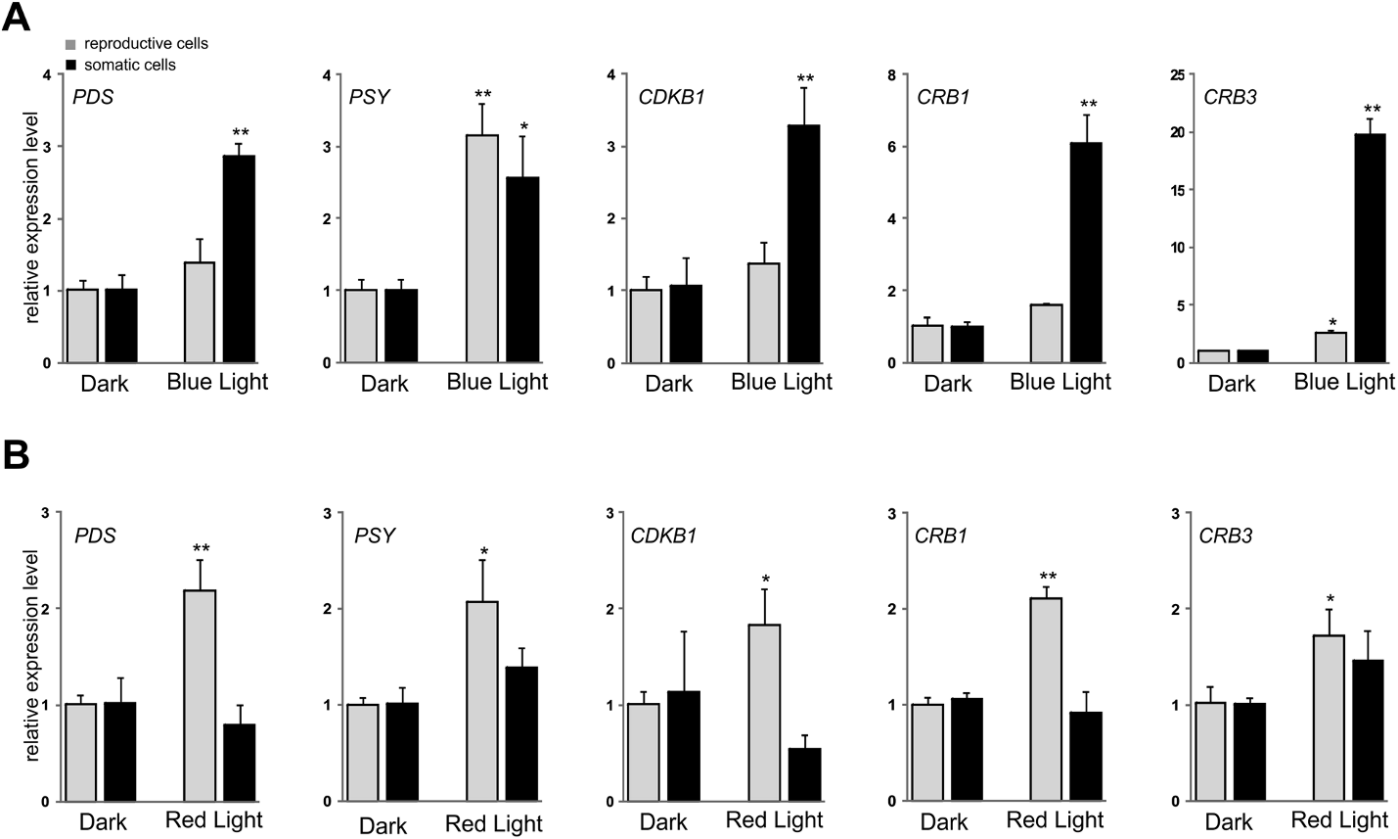
**Blue and red light-induced changes in transcript accumulation of genes encoding cell cycle, clock and carotenoid biosynthesis components.** Cell type separation and light treatments using blue (**A**, 470 nm; 15 μmol photons m^-2^ s^-1^) and red (**B**, 655 nm; 15 μmol photons m^-2^ s^-1^) light were done as described in Figure 2 and Additional file 1: Figure S1A-B. Each experiment was performed in triplicate from at least two different biological samples. The data were averaged and statistically treated (t-test, *P < 0.05; **P < 0.01). Bars show means and standard deviations.

In the closely related alga *Chlamydomonas*, expression of two genes involved in carotenoids biosynthesis pathway, *PDS* and *PSY*, is affected by blue and red light [10, 11, 35]. In the multicellular *Volvox*, the transcript of *PDS* accumulated in each cell type in response to different light quality. Whereas blue light led to increase the transcript level of *PDS* in the somatic cells, red light induced accumulation of this transcript in the reproductive cells (Figure 3A-B). Unlike *PDS*, *PSY* transcript increased in both cell types after blue light irradiation. Red light, however, induced accumulation of *PSY* transcript only in the reproductive cells (Figure 3A-B). We also analyzed the effect of blue and red light on the transcript levels of two subunits of the circadian RNA-binding protein CHLAMY1, i.e., *CRB1* and *CRB2*, which are shown to be associated with circadian clock in green algae [46]. A marked increase in transcript level of *CRB3* (~20-fold) was observed in the somatic cells after exposure to blue light (Figure 3A). *CRB1* also exhibited a relatively high transcript accumulation in these cells following blue light exposure (Figure 3A). In the reproductive cells, however, the transcript level of *CRB1* and *CRB3* were increased moderately in response to blue light. Remarkably, no significant changes in transcript level of these genes have been observed when the somatic cells were exposed to red light (Figure 3B). But in contrast, red light led to modest change in the transcript level of *CBR1* and *CRB3* in the reproductive cells (Figure 3A-B).

Recently, it was shown that the transcript level of *CDKB1*, a plant specific cyclin-dependent kinase which is involved in the regulation of the cell cycle [47, 48], gradually increases following exposure of dark-adapted *Chlamydomonas* cells to red light. However, only modest changes in transcript abundance were observed after irradiation with blue light [10]. In *Volvox*, blue light elevated the transcript level of *CDKB1* only in the somatic cells (Figure 3A). Moreover, a small elevation in the transcript level of *CDKB1* in the reproductive cells was observed following exposure to red light, while no significant change has been observed in the somatic cells (Figure 3B). These results indicated that blue light induces transcript accumulation of the genes involved in circadian clock and cell cycle in the somatic cells, whereas no blue light-induced changes were observed in the reproductive cells.

### Blue and red light induced modulation of *ACDA*and *CA*transcript abundance

**Figure 4:**
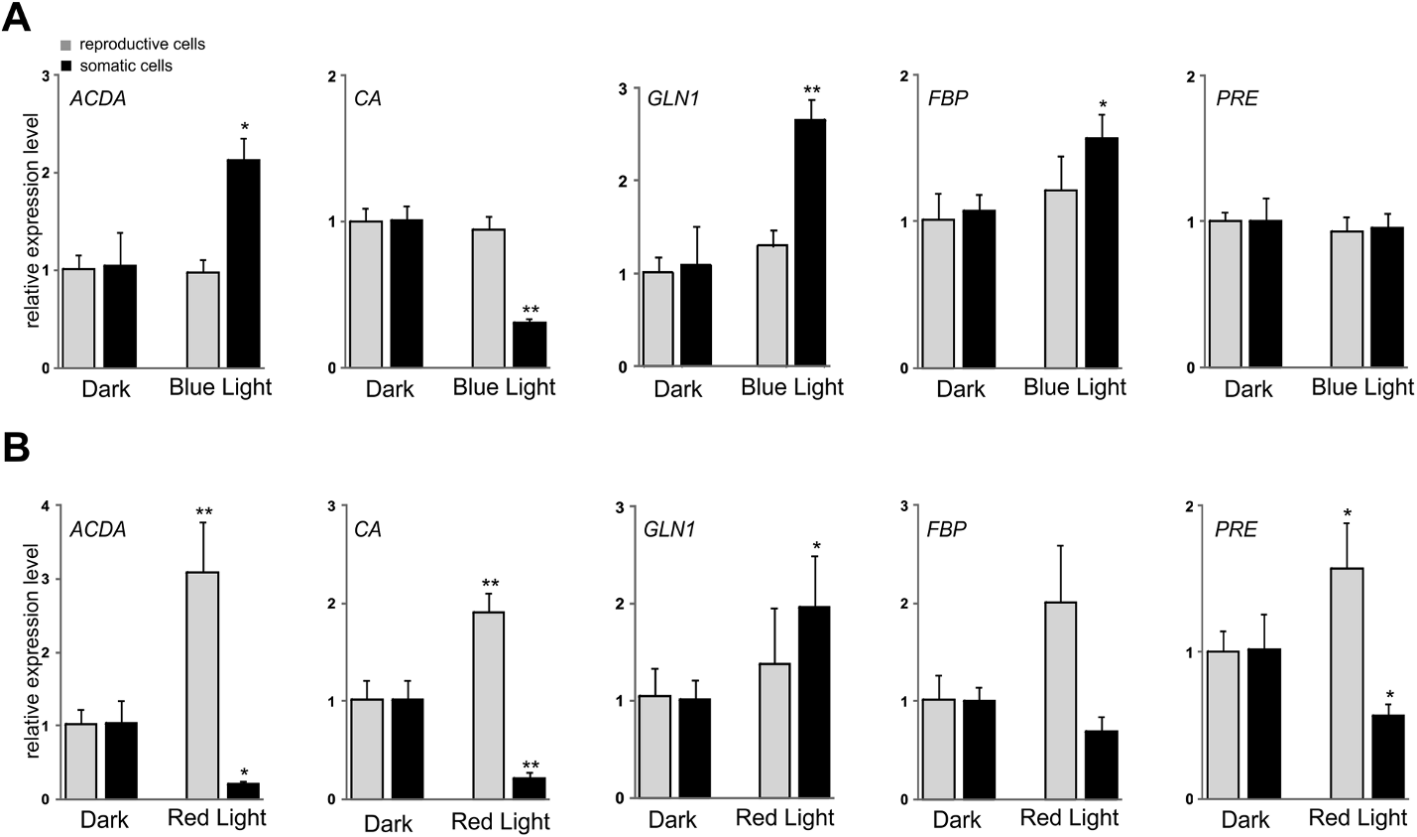
**Blue and red light-induced changes in transcript accumulation of genes encoding components of diverse metabolic pathways.** Cell types were separated as described in Figure 2 and Additional file 1: Figure S1A. After 26 h of dark incubation, the cells were exposed to blue (**A**, 470 nm; 15 μmol photons m^-2^ s^-1^) and red (**B**, 655 nm; 15 μmol photons m^-2^ s^-1^) light for 1 h. Each experiment was performed in triplicate from at least two different biological samples. The data were averaged and statistically treated (t-test, *P < 0.05; **P < 0.01). Bars show means and standard deviations.

Class III guanylyl and adenylyl cyclases represent one of the largest known protein families in the genome of *Chlamydomonas* and *Volvox* [21, 22]. Although these cyclases are not identified so far in plants, they catalyze the synthesis of cGMP and cAMP, which serve as second messengers in a variety of signaling processes, in animals, fungi and prokaryotes [49, 50]. A member of the guanylyl and adenylate cyclase family, i.e., *ACDA*, which has been previously shown to be more expressed in the somatic cells than in reproductive ones [37], was analyzed here. In the somatic cells, blue light led to induce expression of *ACDA*, however, the level was remarkably reduced (~5-fold) following exposure of the cells to the red light (Figure 4A-B). Both blue and red light caused also decreased level of *CA* transcript encoding a zinc-containing carbonic anhydrase in the somatic cells. In contrast, the level of *ACDA* and *CA* transcripts was increased in response to the red light in the reproductive cells (Figure 4A-B).

### Far-red light causes only modest changes in the levels of transcripts from *LHL4*, *PSY*and *ACDA*

Plants make use of red/far-red light-absorbing phytochrome photoreceptors to measure the ratio of red/far-red light that mediate physiological responses by regulating gene expression [51, 52]. Although phytochromes also have been found in charophyta, a division of green algae that includes the closest relatives of the embryophyte plants [53, 54]; no phytochrome genes could be identified in the genomes of volvocine algae *Volvox* and *Chlamydomonas*, even though red- and far-red-regulated gene expression has been observed [6, 10, 11, 33]. As shown in Additional file 6: Figure S5, we could only detect the modest effect of far-red light (735 nm, photon fluence rate of 15 photons m^-2^ s^-1^) on the transcript levels of *LHL4*, *PSY* and *ACDA*. Whereas the transcript level of *LHL4* and *PSY* increased in the somatic cells after far-red light treatments, a reduced level of *ACDA* transcript was observed in both cell types (Additional file 6: Figure S5). These results indicate that the changes in transcript level of genes analyzed in this study are more sensitive to the blue and red light than far-red light. On the other hand, this observation could be interpreted as small support for the existence of a putative far-red-light signaling pathways in the volvocine algae including *Volvox*.

## Discussion

In this study, cell-type specific changes of transcript accumulation of genes associated with various metabolic and cellular pathways in response to the light of different wavelengths were analyzed in *Volvox*. With respect to the cell-type specific photoreceptors, it is very likely that this multicellular alga uses different cell-type specific light signaling pathways to regulate gene expression in a cell-type specific fashion. The results presented here provide an insight into the cell-types specific changes of transcript accumulation which is required to utilize environmental clues such as light towards adequate adaptation of light-affected cellular and developmental processes in each cell type.

### Blue and red light induce quantitative difference of transcript accumulation in both cell types

**Figure 5:**
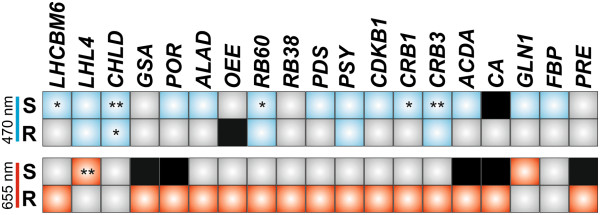
**Cell-type specific light-mediated changes of transcript level.** Enhanced gene expression by blue (470 nm) and red (655 nm) light in the somatic (S) and reproductive (R) cells is highlighted by blue and red, respectively, while repressed expression is shown by black. Grey shows no change in transcript level under given light-treatment. * and ** indicate more than four- and eight-fold increase in transcript level, respectively.

Plants including algae use various kinds of photoreceptors to sense changes in environmental light and to mediate diverse physiological and developmental processes by photoreceptor-mediated orchestrating of gene expression [52, 55]. These light-sensitive proteins allow efficient reprogramming of transcriptome by light-induced activation of transcription factors or regulation of light-induced splicing of target genes [56, 57]. Moreover, tissue or developmentally regulated expression of photoreceptors and/or associated signaling components triggers changes in transcript level of distinct target genes in different organs [58–60]. Considering the fact that the most *Volvox* photoreceptors are expressed in a cell-type specific manner (Figure 1), the main issue to be dealt with is whether this multicellular alga makes use of different distinct light signaling pathways to regulate gene expression in its two entirely different cell types, especially in response to the environmental light. The data presented here show that blue and red light induce cell-type specific changes in the level of transcripts from the most but not all analyzed genes. The transcript of four genes belonging to various functional categories, i.e., *POR*, *PDS*, *CRB1* and *ACDA*, accumulated in the reproductive and somatic cells in response to different light qualities, i.e., red and blue light, respectively (Figure 5). Our data also show that the transcript accumulation in the somatic cells is more sensitive to blue light (14 genes), while red light led to accumulate more transcripts (15 genes) in the reproductive cells (Figure 5). Only *GLN1* and *LHL4* showed increase in transcript levels after red light treatment in the somatic cells. In the closely related alga *Chlamydomonas*, blue light was shown to be more effective for inducing *LHL4* gene expression than red or far-red light [25]. However, although *LHL4* transcript accumulated in both *Volvox* cell types in response to blue light (Figure 2A), red (a marked increase) and far-red (a modest increase) light could elicit elevated expression only in the somatic cells (Figure 2B, Figure 5 and Additional file 6: Figure S5).

In higher plants, a large number of genes are obviously repressed by blue, red and far-red light [9, 61, 62]. Among analyzed genes in this study, only five transcripts were found to be repressed (with a ratio of 0.31-fold or less) in the somatic cells after 1 h of blue and/or red light irradiation; the carbonic anhydrase gene *CA* was down regulated following both blue and red light treatments (Figure 4A-B), while *ACDA, GSA*, *PRE* and *POR* were decreased only in response to red light (Figure 2B and Figure 4B). On the other hand, in the reproductive cells, the transcripts of all these five genes were increased after red light treatment (Figure 5). This indicates that the same light quality causes induction or repression of genes in a cell type-dependent fashion, suggesting that different transcription factors could be involved in downstream red-light-signaling pathway in the two cell types.

### Cell-type specific light signaling pathways behind cellular differentiation

During evolution, the development of complex eukaryotic organisms such as *Volvox* is generated through selective expression of specific fraction of the same genome in different cell types in response to developmental and environmental cues [59, 63]. The two cell types of this multicellular alga represent differential expression pattern of genes from various functional classes [37, 64, 65]. However, little is known regarding cell-type specific changes of gene expression in response to environmental cues including light, which is one of most important environmental signals for controlling growth and development in plants including algae [1, 5, 6, 66, 67]. In plants, for example, blue and red/far-red light are of great importance for the optimization of cellular and developmental processes such as photosynthesis by shade avoidance response and chloroplast positioning [44, 68–70]. Moreover, it is generally assumed that photoreceptors induce light-modulated gene expression to direct appropriate growth and developmental responses [44, 62, 71]. Comparison of the gene expression profiles of the reproductive and somatic cells reported here revealed that distinct cell-type specific light signaling pathways underlying gene expression modulate appropriate transcript regulation in response to light. Thus, different qualities of light appear to trigger distinct cell-type specific changes in transcript level (Figure 5). In particular, blue light increased the transcript level of two photosynthetic relevant components, i.e., *LHCBM6* and *POR*, only in the somatic cells, while those levels were elevated in the reproductive cells by red light (Figure 2A-B). On the other hand, a marked increase in *LHL4* transcript was observed in the somatic cells exposure to red light, while no change was detected in the reproductive cells exposure to the same light quality (Figure 2B). In *Volvox*, regulation of photosynthesis is believed to be the key difference between both cell types. Whereas nuclear genes encode important chloroplast proteins are expressed abundantly in the reproductive cells, repression of their transcription in the somatic cells blocks reproductive activity by preventing cell growth [65, 72, 73]. In *Chlamydomonas*, expression of genes associated with photosynthetic function is affected by blue and red light [23, 25, 33]. Light signaling mediated by phototropin and cryptochrome was shown to be implicated in the induction of gene expression [10, 11]. The observed cell-type specific changes in transcript accumulation of genes associated with photosynthesis in response to light can be traced back to the *Volvox* photoreceptors, which are mostly expressed in a cell-type specific fashion.

In addition, we observed that the transcript of *CRB1* and *CRB3* genes encoding C1 and C3 subunits of RNA-binding protein CHLAMY1, respectively, increased in response to blue light in the somatic cells, but not in the reproductive cells. It has previously been reported that the binding activity of CHLAMY1 changes in a circadian manner [74]. Interaction between CHLAMY1 and these subunits is necessary for RNA-binding activity [75]. Moreover, changes in the level of C1 and C3 affect circadian rhythms in *Chlamydomonas* [46]. A marked increase in the transcript of *CRB3* in the somatic cells after 1 h exposure to blue light (~20-fold) is also consistent with recent report by Beel *et al.* [10] that showed an ~15-fold increase in the C3 transcript after the cells were exposed to 30 min of blue light [10]. However, in contrast, we did not observed any effect of red and far-red light on the transcript level of *CRB3* and *CRB1* in the somatic cells, indicating that blue light is more effective for inducing these clock-relevant components. Therefore, based on these observations in conjunction with a previous study by Iliev *et al.* [46], it is logical to suggest that blue light affect circadian rhythms in a cell-type specific manner. This conclusion is consistent with a recently reported study about cell-type specific circadian rhythms in *Arabidopsis*, which demonstrated that stomatal guard cells have a different period from surrounding epidermal and mesophyll leaf cells [76].

### Biological significance of cell-type specific light signaling pathways

In nature, the *Volvox* populations swim downward to reach cool and dark regions at twilight and then swim back to the euphotic zone at dawn [77]. Because of the wavelength dependent penetration of the light in water [78, 79], changes in detected wavelengths or spectral composition of light could be detected as an environmental signal cue to swim down- or upward at twilight and dawn, respectively, and to regulate developmental and physiological processes for day and night adaptation. Obviously, *Volvox* makes use of a variety of photoreceptors to sense changes in light properties (direction, quality and intensity). Diverse photoreceptors have been shown to be localized within the eyespot of volvocine algae [19, 80, 81], indicating that eyespot and associated structures [5] serve as general sensory organelles to modulate photobehavior, i.e., phototaxis and photophobic responses, and potentially other developmental or adaptive responses. Thus, the eyespot apparatus is required for accurate light-monitoring and light-dependent movement responses to optimize the photosynthetic activities or to avoid photodamages. In *Volvox*, eyespots are exclusively restricted to the somatic cells, in which the most photoreceptor genes are expressed. On other hand, only VR1 is highly expressed in the reproductive cells (Figure 1). Cell-type specific distribution of photoreceptors enables both cell types to optimize cellular and developmental processes differentially in response to environmental light. In addition, light also serves as a time cue that daily resets the circadian rhythms. The light quality, e.g., amount and spectral composition, changes during twilight. For example, twilight is primarily characterized by relative enrichment of the shorter wavelengths (<500 nm) compared to the mid-long wavelengths (500–650 nm) [82]. The ability to sense changes in the light quality (for example, detection of blue light at dawn) triggers signaling pathways towards adjusting cellular processes. However, the marked changes in transcripts of clock relevant components *CRB1* and *CRB3* in the somatic cells in response to only blue light indicate that each cell type has its own genetically predefined circadian rhythm. Moreover, depends on environmental light, physiological and metabolic activities have been shown to be fine-tuned in green algae (reviewed in [1]). In *Chlamydomonas*, it was shown that the activity of carbonic anhydrase (*CA*) is under blue and red light control. The authors could show that photosynthesis is required for regulation of *CA* and, in addition, a blue light stimulated mechanism is also involved in *CA* transcript regulation [39]. Moreover, inhibition studies and mutant analysis have shown that *CA* is important to the function of photosynthesis in *Chlamydomonas* [83]. In *Volvox*, photosynthetic activities seem to be differentially regulated, in the light of morphological and physiological differences between two cell types. Illumination of reproductive cells with red light (which is needed for the photosynthesis-dependent process) led to accumulation of *CA* transcript, while both blue and red light led to reduce its transcript level in the somatic cells (Figure 4). In other words, depending on cell type, light signals could increase or decrease photosynthetic activities. Therefore, it is to assume that distinct light signaling pathways have been evolved to modulate differential regulation of photosynthetic related components in response to environmental light that ensure development and function of specific types of cells.

## Conclusions

The results presented here demonstrate that the multicellular green alga *Volvox* uses different cell-type specific light signaling pathways to modulate gene expression in a cell-type specific manner. This sophisticated gene expression system has been potentially assured through cell-type specific expression of photoreceptors and allows differential regulation of genes involved in various cellular and metabolic pathways in response to environmental light.

## Availability of supporting data

The data sets supporting the results of this article are included within the article and its additional files.

## Electronic supplementary material

Additional file 1: Figure S1: Light treatments of *Volvox* cell types. (PDF 195 KB)

Additional file 2: Figure S2: Spectral distribution and optical characteristics of used LEDs. (PDF 621 KB)

Additional file 3: Table S1: Primer sequences and amplicon characteristics. (PDF 11 KB)

Additional file 4: Figure S3: Cell-type specific transcript analysis of photoreceptor genes 3 h before initiation of cleavage division. (PDF 549 KB)

Additional file 5: Figure S4: Cell-type specific transcript analysis of photoreceptor genes after dark incubation –just before exposure to the test light. (PDF 578 KB)

Additional file 6: Figure S5: Effect of far-red light on cell-type specific transcript accumulation. (PDF 85 KB)
