# Supplementary material for: Cell-type specific light-mediated transcript regulation in the multicellular alga Volvox carteri
Source: BMC Genomics. 2014 Sep 6;15(1):764. doi: 10.1186/1471-2164-15-764 (PMC4167131; doi:10.1186/1471-2164-15-764)
Supplement: Supplementary file 1 — Additional file 1: Figure S1: Light treatments of Volvox cell types. (PDF 195 KB) [file 12864_2014_6442_MOESM1_ESM.pdf]

Supplemental Figure S1:

Light treatments of *Volvox* cell types.

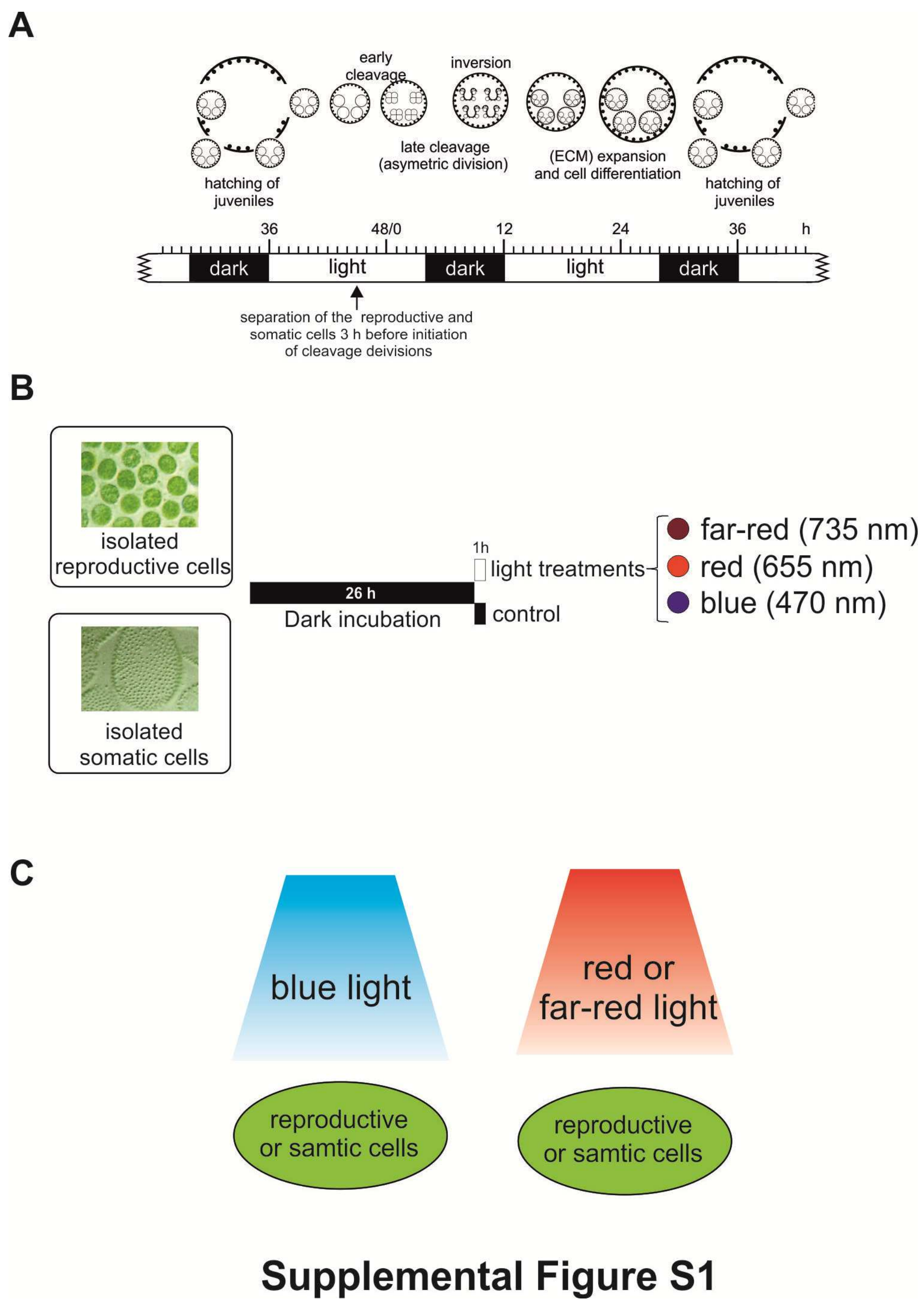

A, *Volvox* wild-type female strain *Eve10* was grown synchronously under vegetative conditions in an 8-h-dark/16-h-light (white light, 100 µmol photons m^{-2} s^{-1}) cycle with a life cycle of 48 h. The reproductive and somatic cells were separated 3 h before initiation of cleavage divisions. **B**, To analyze the effect of different light wavelengths on transcript level in each cell type, isolated cells were transferred to the dark for 26 h before exposure to the test light conditions (15µmol photons m^{-2} $s^{-}1$ of blue, red or far-red lights) for 1 h. The control cells were incubated in the dark. **C**, Schematic illustration of light treatments: The isolated reproductive and somatic cells were placed on peri-dish containing 30 ml *Volvox* standard medium and incubated under blue, red or far-red light.
