# Supplementary material for: Cell-type specific light-mediated transcript regulation in the multicellular alga Volvox carteri
Source: BMC Genomics. 2014 Sep 6;15(1):764. doi: 10.1186/1471-2164-15-764 (PMC4167131; doi:10.1186/1471-2164-15-764)
Supplement: Supplementary file 2 — Additional file 2: Figure S2: Spectral distribution and optical characteristics of used LEDs. (PDF 621 KB) [file 12864_2014_6442_MOESM2_ESM.pdf]

Supplemental Figure S2:

Spectral distribution and optical characteristics of used LEDs.

A

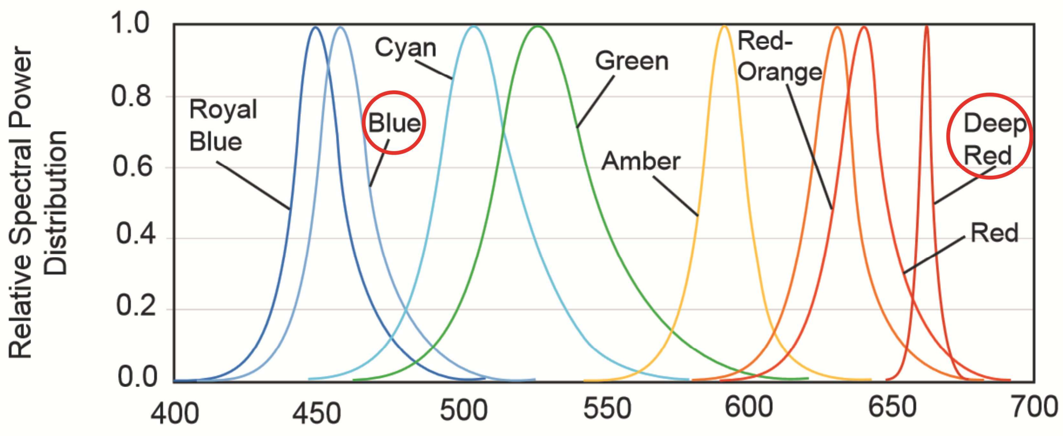

B

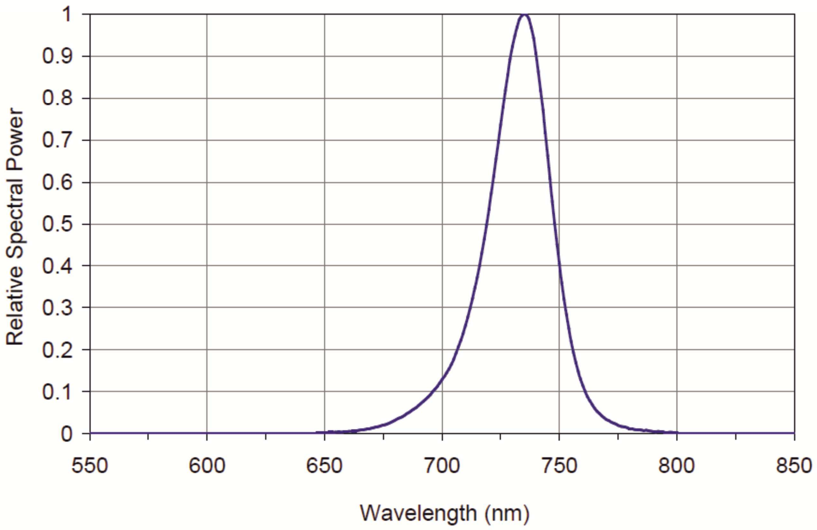

C

Optical characteristics of used LEDs

Color	Minimum wavelength	Typical wavelength	Maximum wavelength
Blue	460.0 nm	470.0 nm	490.0 nm
Deep Red	650.0 nm	655.0 nm	670.0 nm
Far-Red	725.0 nm	735.0 nm	745.0 nm

Supplemental Figure S2

A, Spectral distribution of blue and red LEDs (circled in red). **B**, Spectral distribution of far-red LED. **C**, Wavelength characteristics of used blue, red and far-red LEDs.
