# Supplementary material for: Cell-type specific light-mediated transcript regulation in the multicellular alga Volvox carteri
Source: BMC Genomics. 2014 Sep 6;15(1):764. doi: 10.1186/1471-2164-15-764 (PMC4167131; doi:10.1186/1471-2164-15-764)
Supplement: Supplementary file 3 — Additional file 3: Table S1: Primer sequences and amplicon characteristics. (PDF 11 KB) [file 12864_2014_6442_MOESM3_ESM.pdf]

Supplemental Table S1:

Primer sequences and amplicon characteristics.

Gene	Primer sequences (5´-3´)	Amplicon length (bp) of cDNA	and gDNA	PCR efficiency[a]	R^2	Tm (C°)[b]
LHCBM6	F: GCTTTCGCTCTTGCTACCTCG<br>R: GAGCCACTTAGCACGGTTGG	135	245	99.8	0.998	88.5
CHLD	F: AGCAGCGACTAGTCTGCAATG<br>R: AGACAACCGTGTCGAAACCTC	116	423	99.8	0.989	87
PDS	F: TCTTCATTGCGATGGCGAAGG<br>R: AAGGCCATCTTGGAGCCGTG	119	537	99.8	0.998	87.5
GSA	F: GGAGATAATGAAGATGGTGGCAC<br>R: CTTCTCCAGGTACTCGTAGGC	139	362	99.8	0.988	89
POR	F: GTGCATCATTGTTGGCTCCATC<br>R: AACTCCTGGCCATCCATCATG	138	340	99.8	0.965	89
GLN1	F: CATGCAGGACTTGAGGTCTAAG<br>R: AGGTACACCTCTGAGTCGTG	129	375	99.8	0.987	86.5
CDBK1	F: GACATCAACACTGGGAAGCTG<br>R: GATATGGTTGCTGTCTGAGAGC	128	235	99.8	0.991	83
CRB1	F: TAACCGGTGCTCAGGATGCAG<br>R: TCCACCACTTCGGTTGAACCAAC	127	208	99.8	0.990	82
CRB3	F: GACGCTGCTCGTGAAGTTTGC<br>R: GGATATGTTGCTCAGTCGTGTG	122	874	99.8	0.988	86
ALAD	F: GCAAGAACGTTATTGACTATGTTGCC<br>R: GGATAGTGCGTTGCGACAGAC	144	349	99.8	0.956	86.5
RB60	F: AGCTGGTGTCGGACTACAACG<br>R: TCGAGCTCCTGCAGCTTGTC	118	305	99.8	0.979	85.5
RB38	F: GACGAGGTCCTATCCAACTGG<br>R: CACGGAACGGTCATCCTTGC	120	305	99.8	0.981	86.5
LHL4	F: GTTTCATCGATCGTTGTCGAGTTC<br>R: CACCGAACAGGGCAATCATGG	109	723	99.8	0.997	87.5
FBP	F: GAGCTCTTCACTCTCACAACC<br>R: GCGAGGAAATCTGCTTGCAG	115	282	99.8	0.994	86
PRE	F: TGGACCAGTGCAAGAAGACTG<br>R: CCGTCCATAACGTCTACGTGC	133	240	99.8	0.981	88.5
CA	F: CTGGGATCATGGCTTGCATGG<br>R: TTGCGTTTGGCAAATTGATAGG	110	245	99.8	0.988	84
PSY	F: GACGAACAGACGAGCTGGTG<br>R: GCAGCGTCCAATTCATCGTAG	124	187	99.8	0.984	85
OEE	F: CTTCGCTTCCTTTGCCAATTACC<br>R: TTGGCACGGACCGGCCTGGCAG	111	111	99.8	0.988	88.5
ACDA	F: GTGCCATATGTCGGATTTCTG<br>R: CATGACGATCACGACGTTTC	144	144	99.7	0.992	79
RACK1	F: CACACCAAGGATGTCCTGTC<br>R: CCAATCGTGTACTTGCACTCG	119	355	99.8	0.972	86

F: forward primer, R: reverse primer, gDNA: genomic DNA, cDNA: complementary DNA, R^2: regression coefficient.

[a,b] calculated by Opticon Monitor software (version 1.06, MJ research, Waltham, MA).
