# Supplementary material for: Cell-type specific light-mediated transcript regulation in the multicellular alga Volvox carteri
Source: BMC Genomics. 2014 Sep 6;15(1):764. doi: 10.1186/1471-2164-15-764 (PMC4167131; doi:10.1186/1471-2164-15-764)
Supplement: Supplementary file 4 — Additional file 4: Figure S3: Cell-type specific transcript analysis of photoreceptor genes 3 h before initiation of cleavage division. (PDF 549 KB) [file 12864_2014_6442_MOESM4_ESM.pdf]

Supplemental Figure S3:

**Cell-type specific transcript analysis of photoreceptor genes 3 h before initiation of cleavage division**

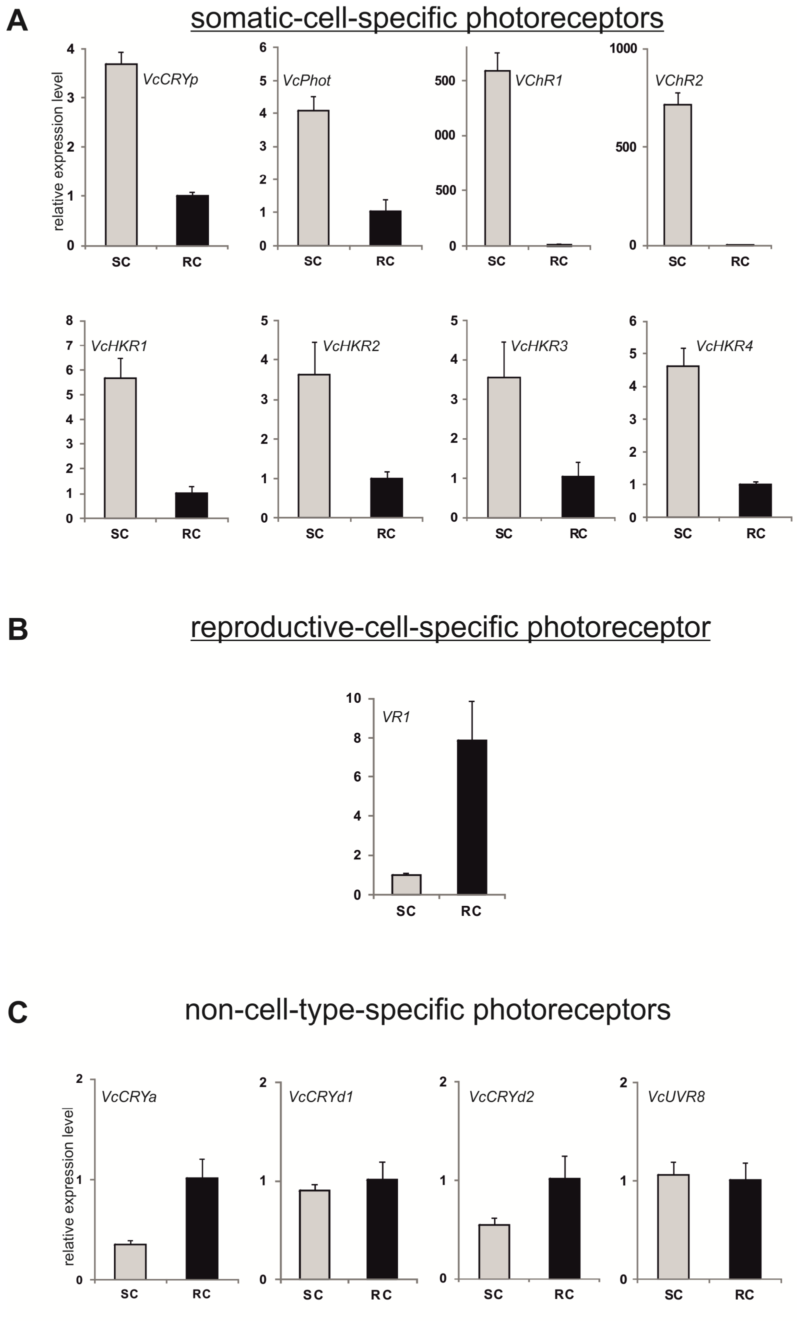


**Supplemental Figure S3**

The reproductive and somatic cells were separated 3 h before initiation of cleavage divisions. The transcript levels were calculated using the $2^{-\Delta\Delta Ct}$ method and *RACK1* as reference genes. The photoreceptors are divided in three groups: somatic-cell-specific photoreceptors (A), reproductive-cell-specific photoreceptors (B) and non-cell-type-specific photoreceptors (C, less than two-fold difference at transcript level between two cell types). Each experiment was performed in triplicate from two different biological samples. The results show the mean and S.D. (error bars). SC, somatic cells; RC, reproductive cells.
