# Supplementary material for: Cell-type specific light-mediated transcript regulation in the multicellular alga Volvox carteri
Source: BMC Genomics. 2014 Sep 6;15(1):764. doi: 10.1186/1471-2164-15-764 (PMC4167131; doi:10.1186/1471-2164-15-764)
Supplement: Supplementary file 5 — Additional file 5: Figure S4: Cell-type specific transcript analysis of photoreceptor genes after dark incubation –just before exposure to the test light. (PDF 578 KB) [file 12864_2014_6442_MOESM5_ESM.pdf]

Supplemental Figure S4:

Cell-type specific transcript analysis of photoreceptor genes after dark incubation -just before exposure to the test light-.

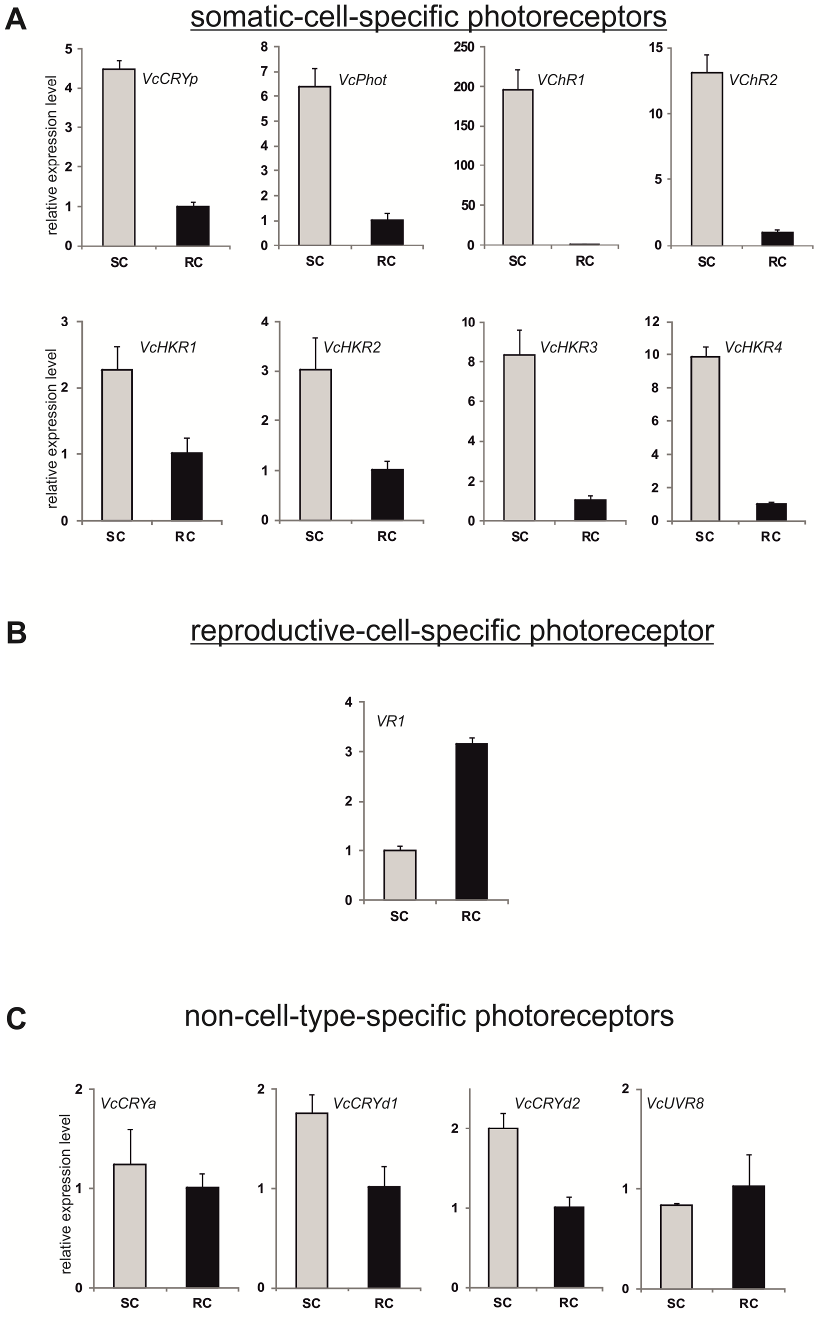

Supplemental Figure S4

The reproductive and somatic cells were separated 3 h before initiation of cleavage divisions and incubated for 26 h in the dark before RNA extraction. The transcript levels were calculated using the $2^{-\Delta\Delta Ct}$ method and *RACK1* as reference genes. The photoreceptors are divided in three groups: somatic-cell-specific photoreceptors (A), reproductive-cell-specific photoreceptors (B) and non-cell-type-specific photoreceptors (C, less than two-fold difference at transcript level between two cell types). Each experiment was performed in triplicate from two different biological samples. The results show the mean and S.D. (error bars). SC, somatic cells; RC, reproductive cells.
