# Supplementary material for: Cell-type specific light-mediated transcript regulation in the multicellular alga Volvox carteri
Source: BMC Genomics. 2014 Sep 6;15(1):764. doi: 10.1186/1471-2164-15-764 (PMC4167131; doi:10.1186/1471-2164-15-764)
Supplement: Supplementary file 6 — Additional file 6: Figure S5: Effect of far-red light on cell-type specific transcript accumulation. (PDF 85 KB) [file 12864_2014_6442_MOESM6_ESM.pdf]

Supplemental Figure S5:

Effect of far-red light on cell-type specific transcript accumulation.

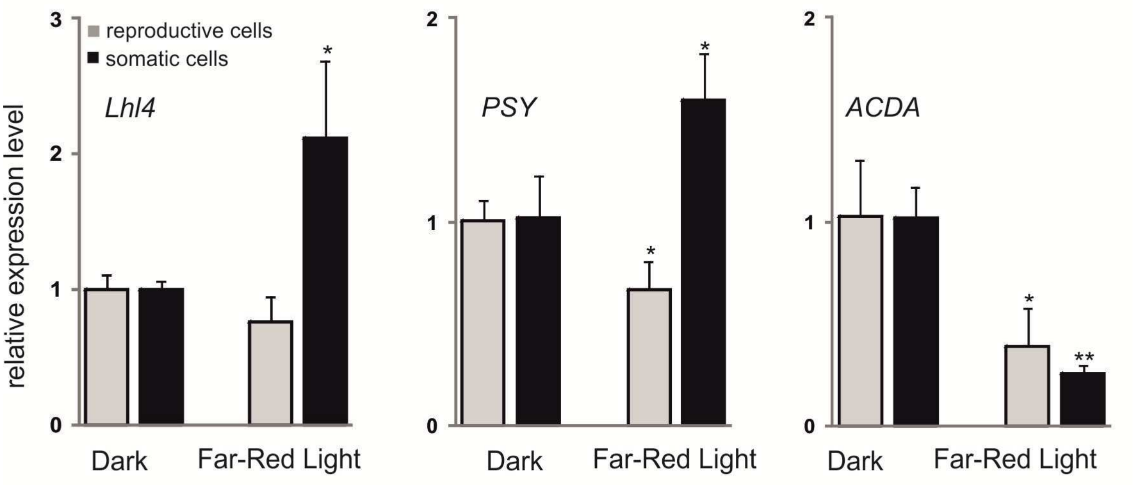

Supplemental Figure S5

Cells were separated and subsequently incubated in the dark as indicated in Figure 2 and Supplemental Figure S1 before exposure to far-red light (735 nm; 15 µmol photons $m^{-2}s^{-1}$) for 1 h. The data were averaged and statistically treated (t-test, $*P < 0.05$; $**P < 0.01$).
